# Perfusion stability in acute stroke: An observational study exploiting repeated CTP imaging

**DOI:** 10.1016/j.ejro.2026.100736

**Published:** 2026-02-17

**Authors:** Alexander Rau, Ömer Bagcilar, Marco Reisert, Horst Urbach, Elias Kellner

**Affiliations:** a Department of Neuroradiology, Medical Center, Faculty of Medicine, University of Freiburg, Freiburg, Germany; b Department of Medical Physics, Medical Center, Faculty of Medicine, University of Freiburg, Freiburg, Germany; c Department of Stereotactic and Functional Neurosurgery, Medical Center, Faculty of Medicine, University of Freiburg, Freiburg, Germany

**Keywords:** CT Perfusion, Demarcation, Infarct Core, Stroke, Temporal Stability

## Abstract

**Background and purpose:**

CT perfusion (CTP) is widely used to assess infarct core in acute stroke, yet real-world data on its reproducibility and temporal dynamics are limited.

**Materials and methods:**

We retrospectively identified patients with repeated CTP scans. Core and hypoperfusion volumes were quantified using standard thresholds (CBF <30 %, Tmax >6 s). Clinical and imaging data were reviewed to identify cases with disruptive events. We analyzed scan-to-scan differences in core volume, hypoperfusion volume, ASPECTS, and intensity metrics, including median Tmax (in hypoperfusion), CBF, and NCCT HU (in core), using Bland-Altman analysis and assessed their association with time between scans.

**Results:**

Among 32 patients with repeated CTP (26 with repeated NCCT), three were excluded due to disruptive events. In the remaining 29 cases, mean scan-to-scan differences for infarct core volume (4.8 ± 19.6 mL), hypoperfusion volume (3.86 ± 39.1 mL), and ASPECTS (–0.4 ± 1.6) indicated minimal systematic bias at the group level but substantial variability. Correlation coefficients were high (r = 0.90, 0.93, and 0.70, respectively; all p < 0.0001), and no statistically significant paired differences or association with scan interval were observed. Intensity-based metrics likewise showed minimal bias with lower variability (Tmax –0.3 ± 1.07 s; CBF –3.11 ± 7.3 %; NCCT HU –3.0 ± 4.9 %), high correlations (Tmax r = 0.87, CBF r = 0.91, NCCT HU r = 0.69; all p < 0.02), and no association with time between scans.

**Conclusions:**

Repeated CTP showed no systematic group-level scan-to-scan bias suggestive of infarct growth, while a substantial degree of variability was observed, with intensity-based metrics demonstrating lower variability than volume estimates. These findings support temporal consistency of perfusion-derived metrics at the group level and question the applicability of linear infarct growth rate (IGR) concepts to perfusion imaging, which primarily reflects a hemodynamic state rather than time-dependent tissue progression.

## 1 Introduction

Computed tomography perfusion (CTP) has become a central component in the evaluation of acute ischemic stroke, offering rapid, quantitative insights into cerebral hemodynamics. It enables estimation of infarct core and penumbral tissue, thereby supporting critical therapeutic decisions such as intravenous thrombolysis and endovascular thrombectomy [1], [2], [3], [4].

Despite its widespread use, only limited data exist on the behavior of perfusion metrics over time, as most patients undergo CTP imaging only once during the acute phase. Repeated CTP scans are rarely performed in clinical practice, primarily due to ethical and logistical constraints. However, in selected scenarios—such as clinical deterioration, technical failures in data transfer, or inter-hospital transfer (“drip-and-ship”) workflows—repeat CTP imaging may occur as part of routine care. Such data offer a unique opportunity to evaluate the repeatability and reproducibility of perfusion imaging, as well as to explore the temporal dynamics of infarct evolution. In particular, they may provide insight into the stability of CTP-derived core and hypoperfusion estimates, the progression of ischemic injury, and the conceptual validity of models such as linear infarct growth rate (IGR) - a topic in which the lack of systematic data has been explicitly noted as a research gap by Ospel et al. [5]. To date, only limited data on short-term repeated CTP is available and indicates stability in either infarct volumes in vessel occlusion [6] or perfusion parameters in internal carotid artery stenosis [7], though a comprehensive evaluation in ischemic stroke is lacking.

In this study, we retrospectively identified a cohort of patients with repeated CTP scans performed as part of clinical stroke care. Using standardized, fully automated perfusion post-processing software, we analyzed scan-to-scan variability in volumetric and intensity-based parameters to assess both the technical reproducibility of CTP and the physiological stability of cerebral perfusion during acute stroke.

## 2 Material and methods

### 2.1 Study design and patient selection

This retrospective study included patients with suspected acute ischemic stroke who underwent repeated CTP imaging within a 10-hour window. The dataset was assembled by querying the institutional PACS system using DICOM C-FIND (FINDSCU) operations to identify patients with two or more CTP acquisitions. Corresponding non-contrast CT (NCCT) scans, if available, were also extracted. Technical metadata, including manufacturer and scanner model, was obtained from the DICOM headers.

### 2.2 Ethics and consent to participate declarations

The study was approved by the Institutional Review Board (BLINDED FOR REVIEW), and informed written consent was waived.

### 2.3 Data acquisition

CTP imaging protocols varied between vendors and clinical sites. Typical scan parameters included a tube voltage of 80 kVp, temporal sampling ranging from 1.5 to 3.5 s, scan duration ranging from 38 to 55 s, and a z-axis coverage of 8–9 cm. Contrast agent was typically administered via a cubital vein using a 16–18 Gauge catheter, with a standard injection protocol of 40 mL iodinated contrast agent delivered at a flow rate of 6 mL/s over 6.6 s, followed by a 30 mL saline flush at 5 mL/s.

The time interval between repeated CTP acquisitions was calculated using the DICOM tag “AcquisitionTime”.

### 2.4 Image analysis

Perfusion imaging was processed using the fully automated software package VEOcore [8] (VEObrain GmbH, www.veobrain.com). The analysis pipeline includes motion correction and denoising, followed by deconvolution of tissue time–attenuation curves with an arterial input function (AIF) using L1-Tikhonov regularization, equivalent to a regularized singular value decomposition formulation. The arterial input function is selected fully automatically: time–attenuation curves are statistically grouped based on peak timing, peak amplitude, and spatial location to distinguish arterial and venous signal pools, candidate curves are ranked according to predefined quality criteria, and the arterial input function is selected accordingly.

The software generates perfusion maps for Cerebral Blood Flow (CBF), Cerebral Blood Volume (CBV), and Time to Maximum (Tmax) of the residue function. CBF and CBV values are normalized to normally perfused tissue defined by Tmax < 4 s. The software also performs automated segmentation and volumetry of hypoperfused tissue and infarct core, based on established thresholds: Tmax > 6 s for hypoperfusion and CBF < 30 % for infarct core [1], [9]. Prior studies have demonstrated good concordance of VEOcore with other widely used perfusion analysis platforms [10], [11].

In addition, VEOcore provides an automated ASPECTS (Alberta Stroke Program Early CT Score) assessment on non-contrast CT images. This is achieved by non-rigidly registering the NCCT to a standardized template space. An anatomical ASPECTS atlas is overlaid, and Hounsfield unit (HU) histograms are generated for each predefined region. A region is flagged as abnormal if the inter-hemispheric difference exceeds a threshold of ΔHU > 2.25, an empirically optimized value based on internal validation.

In addition to volumetric measures, we derived parameter intensity-based metrics by calculating the median value within the above-defined regions: Tmax within the hypoperfusion volume, CBF within the infarct core, and Hounsfield units (HU) from the NCCT within the core. Tmax was measured directly in its native quantitative units (seconds), while CBF and NCCT values were normalized to the median value of the contralateral homologous region.

### 2.5 Visual rating

Disruptive events were defined as clear changes in cerebrovascular status (new occlusion, thrombus migration, or recanalization) and identified in consensus by two experienced neuroradiologists using all available imaging data and clinical records. Cases with such events were excluded from agreement analyses.

In addition, all perfusion datasets underwent a structured technical review to identify potential sources of scan-to-scan variability. This review was performed in consensus by an experienced neuroradiologist and a physicist with specialization in perfusion imaging, with each scan evaluated independently, in randomized order, and blinded to the delay between scans and quantitative results. Assessed factors included completeness and timing of contrast bolus coverage, z-axis coverage, and overall signal quality indicative of measurement noise. This expert consensus review was intended to identify technical contributors to variability rather than to generate subjective ratings; therefore, interobserver agreement statistics were not applicable.

### 2.6 Statistical analysis

All statistical analyses were performed using MATLAB (MathWorks, Natick, MA, USA). Agreement between repeated scans was evaluated using Bland-Altman analyses, Pearson’s correlation coefficients and absolute volumetric differences for infarct core and hypoperfusion. Paired *t*-tests were applied to assess the statistical significance of changes between time points. A two-sided p-value of less than 0.05 was considered statistically significant.

## 3 Results

From 3290 potential stroke cases, we identified 32 patients (15 female, age ranging from 40 to 92 years (median 80 years) with repeated CTP scans (time between scans 0.6–9 h). Of those, 17 patients received intravenous thrombolysis between scans, and none underwent endovascular treatment. Twenty-six patients also had repeated non-contrast CT (NCCT) imaging available. According to DICOM metadata, the first CTP scan was acquired using the following CT systems: Toshiba Aquilion PRIME (n = 7), Siemens SOMATOM Definition AS (n = 5), SOMATOM Definition AS+ (n = 10), SOMATOM Definition Flash (n = 8), SOMATOM Force (n = 2). The second scans were acquired with SOMATOM Definition AS (n = 30) or SOMATOM Definition Flash (n = 2).

**Fig. 1:**
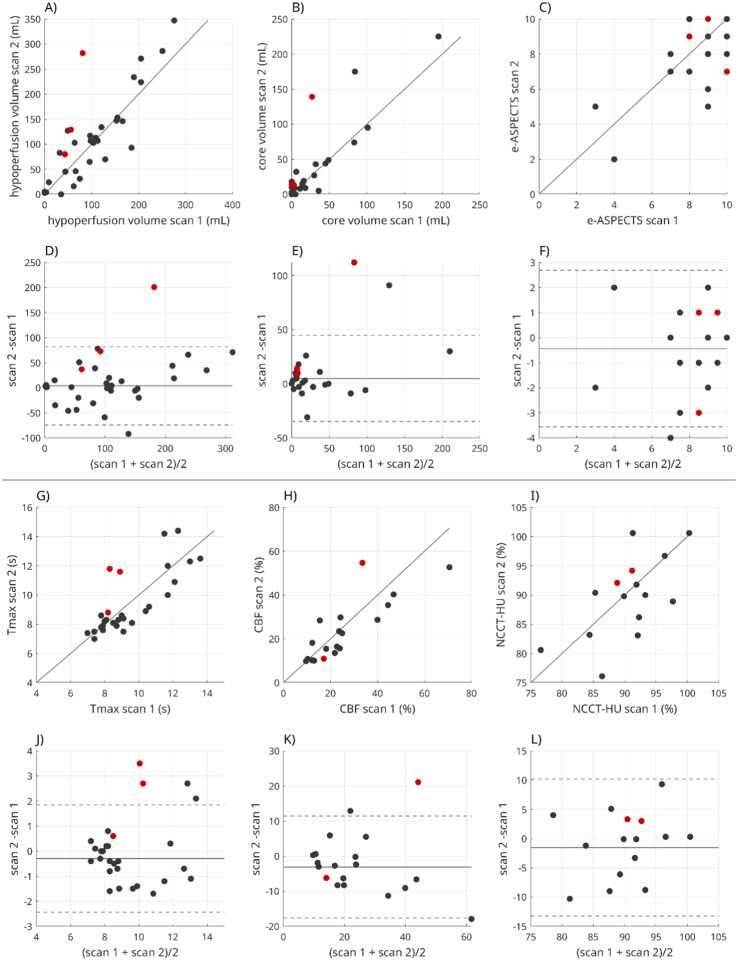
Correlation and Bland–Altman plots for volumetric (A–F) and intensity-based (G–L) metrics. In correlation plots, the solid line indicates the line of identity. In Bland–Altman plots, the solid line indicates the mean difference, and dotted lines indicate the 95 % limits of agreement (mean ± 1.96 SD). Three cases with disruptive events (i.e., additional occlusion or thrombus migration) are highlighted in red and were excluded from the agreement analysis.

**Fig. 2:**
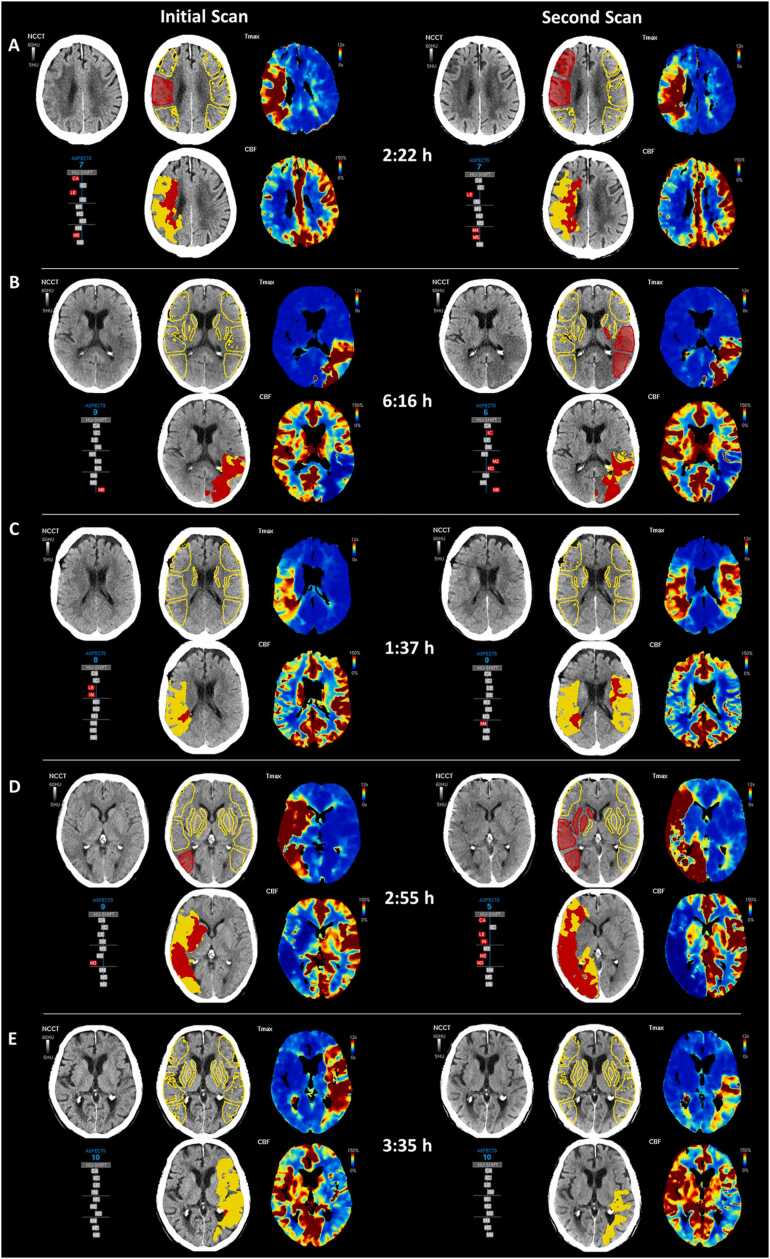
Comparison of repeated CTP scans in exemplary cases. VEOcore software results include perfusion maps (Tmax and CBF), estimated hypoperfusion and infarct core volumes (indicated in yellow and red), and automated ASPECTS overlays highlighting affected regions (regions highlighted in red). Additionally, hemispheric comparisons of Hounsfield unit (HU) shifts are illustrated using gray bar plots. A-B) Perfusion findings are typically stable between scans. In case A, NCCT remains almost unchanged, while in case B, NCCT reveals infarct progression, reflected by a decrease in ASPECTS from 9 to 6. Changes in CTP metrics occurred primarily in presence of disruptive events like additional occlusion (C), or thrombus migration (D).

Perfusion analysis using VEOcore was successful in all 32 cases. Correlations and Bland-Altman plots are illustrated in Fig. 1, and respective outputs of VEOcore in exemplary cases are shown in Fig. 2. Two cases exhibited major changes due to additional vessel occlusions and one due to thrombus migration. These cases were excluded from statistical analysis. No case was recanalized between CTP scans. On technical review of all perfusion scans, no major issues requiring exclusion were identified. However, incomplete or suboptimal bolus capture was observed in 3 scans, differences in z-axis coverage in 2 patients, and increased general measurement noise in 5 scans. These factors were considered potential contributors to the observed scan-to-scan variability.

Agreement between repeated scans was primarily assessed using Bland–Altman analysis. For volumetric perfusion metrics, mean scan-to-scan differences indicated minimal systematic bias, with mean ± standard deviation differences of 4.8 ± 19.6 mL for infarct core volume, 3.86 ± 39.1 mL for hypoperfusion volume, and –0.4 ± 1.6 for ASPECTS. Despite substantial variability, no statistically significant paired differences were observed (p = 0.19 for core volume, p = 0.60 for hypoperfusion volume, and p = 0.20 for ASPECTS). No association was found between the time interval between scans and volumetric differences (p = 0.87, 0.17, and 0.90, respectively).

Correlation coefficients are reported as descriptive measures of linear association and were high for volumetric metrics (r = 0.90 for infarct core volume, r = 0.93 for hypoperfusion volume, and r = 0.70 for ASPECTS; all p < 0.0001), supporting overall concordance between repeated measurements.

For intensity-based measures, Bland–Altman analysis similarly demonstrated minimal bias with lower variability compared with volumetric estimates. Mean ± standard deviation differences were –0.3 ± 1.07 s for Tmax within the hypoperfusion volume, –3.11 ± 7.3 % for CBF within the infarct core, and –3.0 ± 4.9 % for NCCT Hounsfield units within the core. No statistically significant paired differences or associations with scan interval were observed (p = 0.92 for Tmax, p = 0.28 for CBF, and p = 0.18 for NCCT HU). Corresponding correlation coefficients were r = 0.87 for Tmax, r = 0.91 for CBF, and r = 0.69 for NCCT HU (all p < 0.02).

## 4 Discussion

This study retrospectively investigated patients who received repeated cerebral perfusion imaging to investigate reproducibility and stability of derived metrics. The findings can be interpreted from both technical and clinical perspectives.

From a technical standpoint, our data indicate that repeated CTP does not exhibit a systematic scan-to-scan bias suggestive of infarct growth, supporting temporal consistency of perfusion-derived metrics at the group level. However, a substantial degree of variability was observed at the individual level, particularly for automated threshold-derived volumetric measures. Although visual quality control did not reveal major technical failures such as severe motion artifacts, it is well recognized that perfusion imaging is subject to multiple sources of variability [12], [13], including noise amplification, deconvolution instability, and, in particular, sensitivity to hard thresholding, which can amplify small fluctuations in perfusion values. This interpretation is further supported by the lower variability observed for intensity-based measures compared with threshold-derived volumes. The magnitude of variability observed in this study (SD ≈ 20 mL for infarct core and ≈ 40 mL for hypoperfusion volume) is comparable to that reported in prior studies comparing different CTP software packages applied to the same raw data [14], [15], [16], suggesting that much of the observed dispersion likely reflects intrinsic measurement variability rather than true biological change.

While prior data have demonstrated the stability of CTP metrics in patients with internal carotid artery stenosis [7], our results extend this finding to cases with vessel occlusion and show no evidence of metric deterioration suggestive of collateral failure or progressive perfusion compromise.

These findings are also consistent with other prior investigations on repeated perfusion imaging. Lin et al. conducted repeated CTP scans approximately 24 h apart, in a cohort where most patients received intravenous thrombolysis but no mechanical thrombectomy [17]. They reported stable values for CBV, CBF, MTT, and Tmax in brain regions without corresponding symptoms, with major perfusion differences observed primarily in areas affected by reperfusion. The robustness of perfusion parameters over time has also been explored in MRI-based studies. For instance, it was demonstrated that in patients with large diffusion–perfusion mismatch who were not treated with thrombolysis, both the diffusion-weighted imaging-derived and perfusion MRI-derived infarct volumes remained stable between initial and 4-hour follow-up scans [18]. Preclinical studies further corroborate these findings. In a canine stroke model, Harris et al. observed that some brain regions maintained stable hypoperfusion over several hours, while other areas exhibited delayed perfusion decline—highlighting the distinction between stable infarct core and evolving penumbral zones [19]. Similarly, Duong et al. demonstrated reproducibility of perfusion and diffusion metrics across imaging sessions in various experimental settings [20]. Additionally, Seners et al. investigated inter-hospital infarct growth using MRI and found that in a subset with repeated perfusion-weighted imaging, the hypoperfusion intensity ratio remained stable, supporting the temporal robustness of perfusion imaging [21]. Collectively, these studies—along with our results—support the notion that perfusion imaging-derived infarct quantification is generally stable over time.

Finally, our findings closely mirror those of Valente et al., who found that perfusion core growth during transfer was uncommon and mainly associated with lower ASPECTS at baseline [6]. While their analysis focused on volumetric core changes, we additionally examined intensity-based variability, providing a finer-grained view of perfusion stability.

Due to the limited availability of repeated imaging with the same modality, some studies have compared sequential scans acquired using different modalities. For Instance, Wouters et al. investigated infarct growth during interhospital transfer and reported significant changes [22]. However, their analysis compared baseline CTP with follow-up MRI-DWI, which measure fundamentally different aspects of stroke pathophysiology. However, such comparisons likely reflect differences in modality-specific imaging features rather than true longitudinal changes, and should be interpreted with caution.

These findings have important implications for the increasingly discussed concept of infarct growth rate (IGR), which is typically defined as the infarct core volume divided by the time from symptom onset—implicitly assuming a linear progression of ischemic injury. While this model may hold intuitive appeal, particularly in the context of non-contrast CT, where visible hypodensity generally corresponds to irreversible tissue damage [23], [24], [25], its application to perfusion imaging [23], [26], [27], [28] appears conceptually problematic.

A recent commentary by Pensato et Ospel [29] supports this perspective, emphasizing that CTP reflects the current hemodynamic state rather than established infarction, and therefore should not be used to infer infarct growth rate. Although a related study by the same group [30] reported associations between infarct progression and perfusion-derived metrics—particularly the hypoperfusion intensity ratio—true volumetric infarct growth was not directly assessed. Rather, HIR likely serves as a probabilistic indicator of tissue outcome.

Taken together, these insights reinforce the view that perfusion imaging reflects a dynamic risk state rather than deterministic lesion expansion, making linear IGR models conceptually incompatible with its physiological basis. CTP captures real-time cerebral hemodynamics, with perfusion deficits reflecting potentially reversible ischemia influenced by collateral status and reperfusion, rather than steady volumetric growth. Interpreting CTP-derived core as a continuously expanding entity oversimplifies stroke pathophysiology. In our cohort, we observed stable perfusion profiles across repeated scans despite progression on NCCT (Fig. 2B), suggesting that NCCT changes likely reflect evolving edema and increasing lesion conspicuity, while perfusion-based core estimates may remain stable in the absence of hemodynamic shifts.

Our study has several limitations. While a key strength is the consistent use of the same imaging modality across time points, the overall sample size remains modest, which may limit generalizability. Moreover, the retrospective design introduces a potential selection bias, as repeated CTP imaging was likely performed in the absence of clinical improvement or even deterioration rather than as part of a standardized protocol. The cohort also included a mix of patients who did and did not receive intravenous thrombolysis, potentially introducing treatment-related heterogeneity. Despite these limitations, our observational data offer valuable preliminary insights that may help inform the planning and design of future prospective trials. Given the ethical and logistical challenges associated with repeated imaging in acute stroke patients—particularly outside of standard clinical indications—our findings can provide a useful reference point for future investigations into infarct evolution and the temporal stability of perfusion imaging.

In summary, our analysis indicates absence of systematic group-level bias of automated CTP metrics across repeated acquisitions in real-world clinical settings, without systematic scan-to-scan bias. At the same time, residual variability was observed at the individual level, particularly for threshold-derived volumetric measures, emphasizing the need for cautious interpretation and awareness of the inherent variability of automated volume estimates. In vessel occlusion, perfusion parameters showed temporal stability, challenging the direct application of the infarct growth rate concept and supporting a more differentiated, probabilistic view of infarct evolution.

## CRediT authorship contribution statement

**Alexander Rau:** Writing – review & editing, Writing – original draft, Visualization, Methodology, Investigation, Formal analysis, Data curation. **Marco Reisert:** Writing – review & editing, Supervision, Software, Resources, Methodology. **Ömer Bagcilar:** Writing – review & editing, Investigation, Data curation. **Elias Kellner:** Writing – review & editing, Writing – original draft, Visualization, Validation, Supervision, Software, Resources, Project administration, Methodology, Investigation, Data curation, Conceptualization. **Horst Urbach:** Writing – review & editing, Validation, Supervision, Software, Resources, Data curation.

## Ethical statement

The study was approved by the Institutional Review Board (Ethics Committee – University of Freiburg; EK 20/1047), and informed written consent was waived.

## Funding

This research did not receive any specific grant from funding agencies in the public, commercial, or not-for-profit sectors.

## Declaration of Competing Interest

The authors declare the following financial interests/personal relationships which may be considered as potential competing interests. Elias Kellner reports a relationship with VEObrain that includes: consulting or advisory and employment. If there are other authors, they declare that they have no known competing financial interests or personal relationships that could have appeared to influence the work reported in this paper.

## Data Availability

Data and code are available from the authors upon reasonable request and approval by the local ethics committee.
